# *Hsp90* Gene Is Required for *Mi-1*-Mediated Resistance of Tomato to the Whitefly *Bemisia tabaci*

**DOI:** 10.3390/plants12030641

**Published:** 2023-02-01

**Authors:** Susana Pascual, Clara I. Rodríguez-Álvarez, Isgouhi Kaloshian, Gloria Nombela

**Affiliations:** 1 Entomology Group, Plant Protection Department, National Institute of Agricultural and Food Research and Technology (INIA), Spanish National Research Council (CSIC), Ctra. Coruña km 7, 28040 Madrid, Spain; 2 Department of Plant Protection, Institute for Agricultural Sciences (ICA), Spanish National Research Council (CSIC), Serrano 115 Dpdo., 28006 Madrid, Spain; 3 Department of Nematology, University of California, Riverside, CA 92521, USA

**Keywords:** *Bemisia tabaci*, HSP90, insects, Mi-1, pest resistance, tomato, VIGS, whiteflies

## Abstract

The *Mi-1* gene of tomato (*Solanum lycopersicum*) confers resistance against some nematodes and insects, but the resistance mechanisms differ depending on the harmful organism, as a hypersensitive reaction (HR) occurs only in the case of nematodes. The gene *Rme1* is required for *Mi-1*-mediated resistance to nematodes, aphids, and whiteflies, and several additional proteins also play a role in this resistance. Among them, the involvement of the chaperone HSP90 has been demonstrated in *Mi-1*-mediated resistance for aphids and nematodes, but not for whiteflies. In this work, we studied the implication of the *Hsp90* gene in the *Mi-1* resistance against the whitefly *Bemisia tabaci* by means of Tobacco rattle virus (TRV)-based virus-induced gene silencing (VIGS). The silencing of the *Hsp90* gene in tomato Motelle plants carrying the *Mi-1* gene resulted in a decrease in resistance to whiteflies, as oviposition values were significantly higher than those on non-silenced plants. This decrease in resistance was equivalent to that caused by the silencing of the *Mi-1* gene itself. Infiltration with the control TRV vector did not alter *Mi-1* mediated resistance to *B. tabaci*. Similar to the *Mi-1* gene, silencing of *Hsp90-1* occurs partially, as silenced plants showed a significant but not complete suppression of gene expression. Thus, our results demonstrate the requirement of *Hsp90* in the *Mi-1*-mediated resistance to *B. tabaci* and reinforce the hypothesis of a common model for this resistance to nematodes and insects.

## 1. Introduction

*Bemisia tabaci* (Hemiptera: Aleyrodoidea) is one of the most damaging agricultural pest species worldwide, with a high polyphagia, virus transmission capacity, and insecticide resistance problems [1,2,3]. *B. tabaci* is a complex of cryptic species [4,5], with MED and MEAM being two of the most damaging ones, formerly known as biotypes B and Q, respectively [6]. Higher fitness of biotype Q in the context of intensive insecticide use has resulted in frequent competitive displacement of biotype B in many locations, including Spain [7,8,9]. Problems associated with traditional chemical pest control make plant resistance an essential tool for the integrated pest management of *B. tabaci*. Although sources of resistance can be found in the wild relatives of cultivated plants, so far, the only cloned resistance (*R*) gene against whiteflies is the tomato *Mi-1* gene [10]. This gene also provides resistance to root-knot nematodes (*Meloidogyne* spp.) and other phloem feeding insects such as the potato aphid, *Macrosiphum euphorbiae*, and the tomato psyllid *Bactericerca cockerelli* [11,12,13,14]. *Mi-1*-mediated resistance works by following a gene-for-gene interaction, resulting in a hypersensitive reaction (HR) in the case of nematodes, but not in the case of insects [15]. Factors in the epidermis and/or mesophyll of *Mi-1*-resistant tomato inhibit the whiteflies from reaching the phloem sieve elements [16], which results in reduced host suitability and whitefly reproduction [17].

The *Mi-1* gene was introduced into cultivated tomato from the wild relative *Lycopersicum peruvianum* [18] and is present in many tomato cultivars [19]. Like other *R* genes, *Mi-1* encodes a coiled-coil domain nucleotide binding site leucine-rich repeat (CC-NLR) protein [20,21]. Previous research has demonstrated the role of salicylic acid (SA) in *Mi-1*-mediated resistance to whiteflies [22]. More recently, the baseline differences in the tomato transcriptomic profile associated with the *Mi-1* gene and the changes after infestation with *B. tabaci* were investigated [23]. Among other results, the differential gene expression provided support to the fact that the attack of whiteflies does not promote HR in tomato leaves, as demonstrated for aphids in tomato [15] or whiteflies in Arabidopsis [24]. The *Rme1* gene is also required for *Mi-1*-mediated resistance to nematodes, aphids, and whiteflies, acting early in the *Mi-1* pathway, either at the same step as the *Mi-1* product or earlier in the response cascade [25]. However, knowledge on the genes implicated in *Mi-1*-mediated resistance to whiteflies is not complete. In the case of nematodes and aphids, *Mi-1*-mediated resistance involves several additional genes [26,27], among which are *Sgt1* and *Hsp90*, this last gene encoding the chaperone heat shock protein 90 or HSP90 [25,28,29]. Moreover, the heat shock factor HsfA1a is essential for *Mi-1*-mediated nematode resistance [30].

The chaperone HSP90 is highly conserved in most living organisms [31] and it is involved in diverse processes, such as protein folding and degradation and signal transduction [32,33,34]. In *Nicotiana benthamiana*, HSP90 plays important roles in plant growth and development [35]. In tomato, moderate heat stress enhances the strength of jasmonic acid (JA) responses through the activity of HSP90 [36], and overexpression of *Hsp90.2* gene resulted in significant increases in root biomass and architecture, as well as tolerance to salinity and drought stresses [37]. Regarding biotic stress, HSP90 is required to activate the HR during non-host resistance in different pathosystems [31].

Most of the R proteins form complexes with other plant proteins, such as HSP90, through which they detect the effectors of the attacking organisms [38]. HSP90 associates with additional proteins, RAR1 or/and SGT1, stabilizing R proteins to keep them in a controlled and competent conformation state in order to detect pathogen signals [31]. RAR1 and SGIT1 are HSP90 co-chaperones in many pathosystems [35,39,40,41,42,43,44,45]. However, this is not universal as tomato *I-2* gene-mediated resistance to *F. oxysporum* requires the chaperone HSP90 and protein phosphatase 5 (PP5), but does not require SGT1 or RAR1 [46]. In rice and soybean, HSP90 interacts with the co-chaperone Hop/Sti [47,48]. In *Mi-1*-mediated resistance to aphids and nematodes, HSP90 interacts with SGT1 but not RAR1. It is hypothesized that nematode and insect Avr proteins would modify RME1, triggering a conformational change and the formation of HSP90, SGT1, and *Mi-1* signalosome that would activate a defence signalling pathway [28].

The virus-induced gene silencing (VIGS) technique was used to demonstrate the involvement of *Hsp90* in *Mi-1-*mediated resistance to aphids and nematodes [28]. Numerous studies on the role of *Hsp90* in plant stress resistance used VIGS too [35,39,41,44,45,49,50,51,52,53]. VIGS is a post-transcriptional gene silencing (PTGS) method used by plants as a defence mechanism against invading viruses [54]. It is an easy affordable technique to study functional genomics in many plants including Solanaceae such as *N. benthamiana* and tomato [54]. TRV is the choice for a VIGS vector because of the high susceptibility to this virus of a wide range of hosts, with mild viral symptoms [55,56]. TRV is a positive-strand RNA virus with a bipartite genome, which can move systemically in many plants, first being used for endogenous gene silencing in *N. benthamiana* [57]. TRV-VIGS has also been used in many other plant species, including most dicotyledonous plants, some monocotyledonous plants, and even some trees [54].

Although the involvement of *Hsp90* in *Mi-1*-mediated resistance of tomato to aphids and nematodes was previously demonstrated by silencing of that gene in carrying *Mi-1* plants (Motelle cultivar) [28], its potential implication in such a resistance to whiteflies was unknown. The same methodology (TRV-based VIGS) was used now to evaluate the involvement of *Hsp90* in the *Mi-1*-mediated resistance to whitefly *B. tabaci*, which is the objective of this work.

## 2. Results

### 2.1. Evaluation of the Effects of Agroinfiltration with the Vector TRV

**Figure 1:**
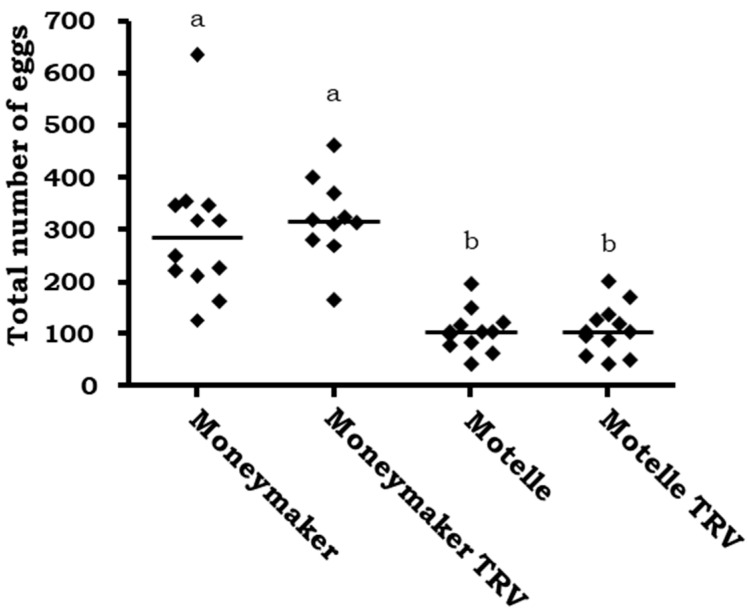
Number of eggs per leaflet on plants of the cultivars Moneymaker (*mi-1/mi-1*) and Motelle (*Mi-1*/*Mi-1*), infiltrated or not with the empty pTRV1:pTRV2 vector (TRV) and infested with *B. tabaci* females. In each of the three independent assays, four plants were used for each tomato genotype and treatment. Each dot represents the mean number of eggs counted on three leaflets from a single plant. The lines indicate the median values of 12 independent plants. Different letters indicate significant differences (*p* < 0.05) by the non-parametric Kruskal–Wallis test and Dunn’s post-test.

Motelle and Moneymaker, the resistant and susceptible tomato cultivars, respectively, were agro-infiltrated with the TRV-VIGS vector and infested with *B. tabaci* in a non-choice assay to test the effect of TRV vector on the whitefly fecundity. The results of these tests are shown in Figure 1. Considering each tomato cultivar separately, the number of eggs on the plant leaflets was similar, regardless of infiltration or not with the “empty” pTRV1:pTRV2 vector (TRV). No statistically significant differences due to TRV infiltration were found for either Moneymaker (*p* > 0.05) or Motelle (*p* > 0.05) plants. On the contrary and as expected, significant differences were obtained in the number of eggs on the control (not infiltrated) plants of the cultivars Moneymaker and Motelle (*p* < 0.001). The difference between Moneymaker and Motelle was maintained after infiltration with the empty TRV vector (*p* < 0.05).

**Figure 2:**
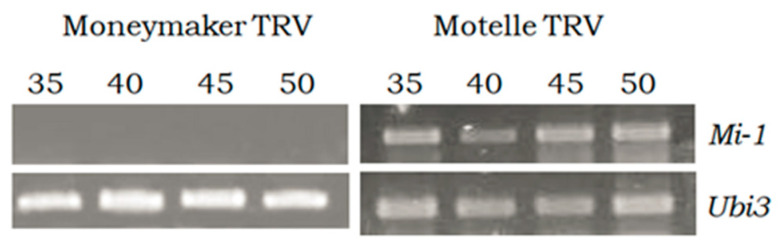
Relative expression levels of the *Mi-1* gene obtained by RT semi-quantitative PCR in Moneymaker (*mi-1*/*mi-1*) and Motelle (*Mi-1*/*Mi-1*) plants infested with *B. tabaci* and infiltrated with the vector pTRV1:pTRV2 empty (TRV). The ubiquitin gene (Ubi3) was used as an endogenous control. The PCR cycles (35, 40, 45, 50) used are indicated.

The expression of the *Mi-1* gene was evaluated by RT semi-quantitative PCR analysis in the agroinfiltrated plants. The results of the semi-quantitative analysis of the relative expression of the *Mi-1* gene after infestation by *B. tabaci* in Motelle and Moneymaker plants infiltrated with the empty TRV vector are shown in Figure 2. No transcript expression was obtained in Moneymaker plants (lacking the *Mi-1* gene). In Motelle plants, the expression of the *Mi-1* gene was clearly observed from cycle 35 of the PCR. As the semi-quantitative PCR analysis clearly shows the differences in expression of the *Mi-1* gene between the cultivars Motelle and Moneymaker, it was not necessary to quantify such expression by qPCR.

### 2.2. Mi-1 Gene Silencing in Tomato Plants

**Figure 3:**
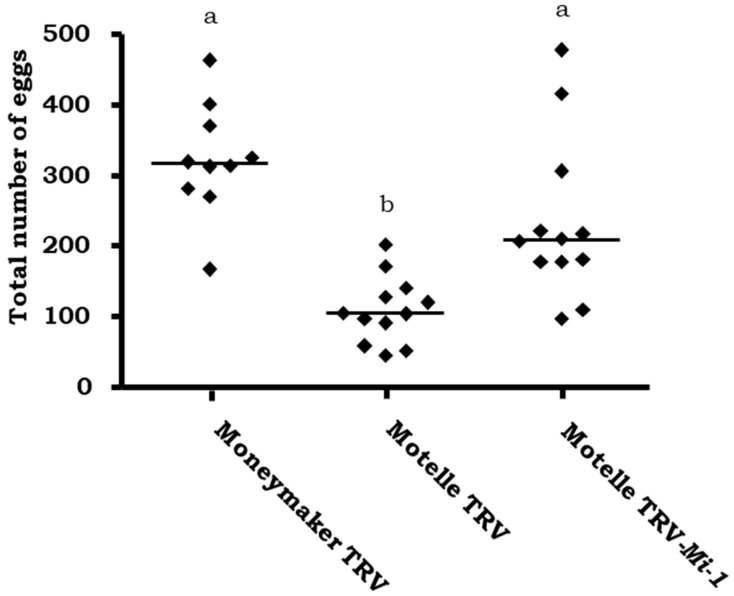
Number of eggs per leaflet on plants of the cultivars Moneymaker (*mi-1/mi-1*) and Motelle (*Mi-1/Mi-1*), infiltrated with the empty pTRV1:pTRV2 vector (TRV) and on Motelle plants agroinfiltrated with the vector pTRV1:pTRV2-Mi (Motelle TRV-Mi) and infested with *B. tabaci* females. In each of the three independent assays, four plants were used for each tomato genotype and treatment. Each dot represents the mean number of eggs counted on three leaflets from a single plant. The lines indicate the median values of 12 independent plants. Different letters indicate significant differences (*p* < 0.05) by the non-parametric Kruskal–Wallis test and Dunn’s post-test.

Tomato cultivar Motelle plants were agroinfiltrated with TRV-*Mi* and infested with *B. tabaci* in a non-choice assay and whitefly fecundity was evaluated. The number of eggs was counted on the leaflets of Motelle plants infiltrated with TRV-*Mi* and values were compared to those on Motelle and Moneymaker plants infiltrated with the empty vector TRV. These results are shown in Figure 3. Significant differences (*p* < 0.05) were obtained between Motelle plants infiltrated with the empty vector TRV and Motelle plants infiltrated with the TRV-*Mi* vector. This demonstrates that silencing of the *Mi* gene decreases resistance against whiteflies, until similar levels to that of the highly susceptible cultivar Moneymaker infiltrated with the empty vector.

**Figure 4:**
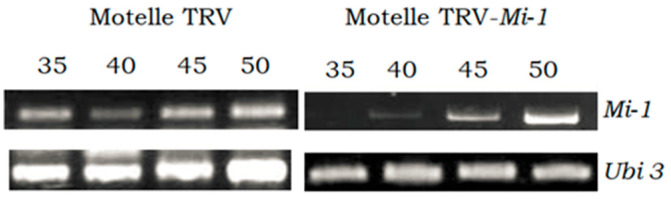
Expression levels of the *Mi-1* gene obtained by RT semi-quantitative PCR in leaflets of Motelle (*Mi-1/Mi-1*) plants infiltrated with the empty vector (Motelle TRV) and leaflets of plants agroinfiltrated with the vector pTRV1:pTRV2-Mi (Motelle TRV-*Mi*), infested with *B. tabaci* females. The ubiquitin gene (*Ubi3*) was used as an endogenous control. The PCR cycles (35, 40, 45, 50) used are indicated.

**Figure 5:**
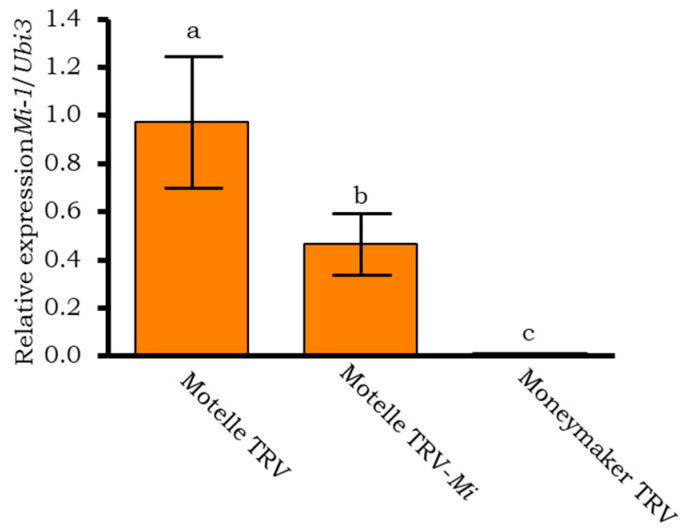
Expression of *Mi-1* in agroinfiltrated tomato plants using RT-qPCR analysis. The expression of the *Mi-1* gene was analysed in leaflets of Motelle (*Mi-1/Mi-1*) and Moneymaker (*mi-1/mi-1*), plants agroinfiltrated with the empty vector pTRV1:pTRV2 (TRV), and in leaflets of Motelle plants agroinfiltrated with the vector pTRV1:pTRV2-Mi (Motelle TRV-*Mi*), two days after infestation with *B. tabaci* females. Each bar is the mean ± standard error of *Mi-1* expression in two biological samples and three technical replicates, normalized with the expression of the endogenous ubiquitin gene (*Ubi3*). Different letters indicate significant differences (*p* < 0.01) by Tukey’s test.

**Figure 6:**
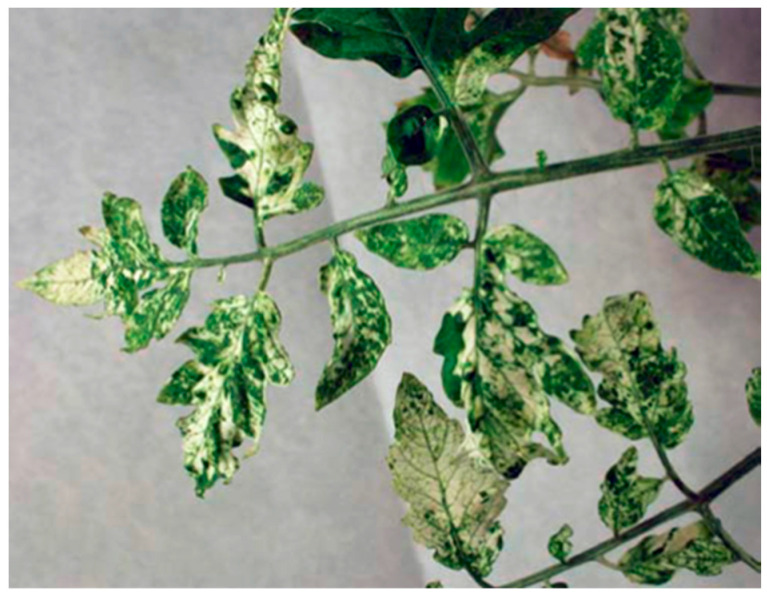
Typical irregular and patchy photobleaching symptoms obtained by silencing of the *PDS* gene in tomato leaves. Some leaflets show a virtually complete photobleaching while others have green areas.

To confirm silencing of the *Mi-1* gene, its expression was analyzed by semi-quantitative (Figure 4) and real-time quantitative PCR (Figure 5). Only the Motelle plants were analyzed by semi-quantitative PCR as no expression of the gene was previously obtained in Moneymaker plants (Figure 2). Figure 4 shows that, in the leaflets of Motelle TRV-*Mi* plants (which had a large number of eggs), the expression of the *Mi-1* gene was not clearly detected until cycle 45, while in Motelle plants infiltrated with the empty vector (Motelle TRV), *Mi-1* expression was detected from cycle 35. This indicates a partial silencing of *Mi-1* expression; namely, the expression values of *Mi-1* do not reach zero because its silencing occurs only in a subset of the cells of the analysed tissue, as it is observed in the mosaic pattern of photobleaching when silencing the phytoene desaturase (*PDS*) gene (Figure 6).

The expression of the *Mi-1* gene in leaflets of the above-mentioned Motelle plants was also compared by RT-qPCR (Figure 6). Moneymaker TRV plants were included as a control. Significantly (*p* = 0.001) lower levels of expression of *Mi-1* were obtained in the leaflets from Motelle TRV-*Mi-1* agroinfiltrated plants than in those from Motelle TRV plants. Significant differences (*p* = 0.002) were also obtained in the comparison between Motelle TRV-*Mi* and Moneymaker TRV control plants. Although gene expression was reduced in *Mi-1* silenced plants, these levels did not reach zero, as in Moneymaker TRV plants, indicating a partial silencing of the *Mi-1* gene as demonstrated in the semiquantitative PCR analysis.

### 2.3. Hsp90-1 Gene Silencing in Tomato Plants

**Figure 7:**
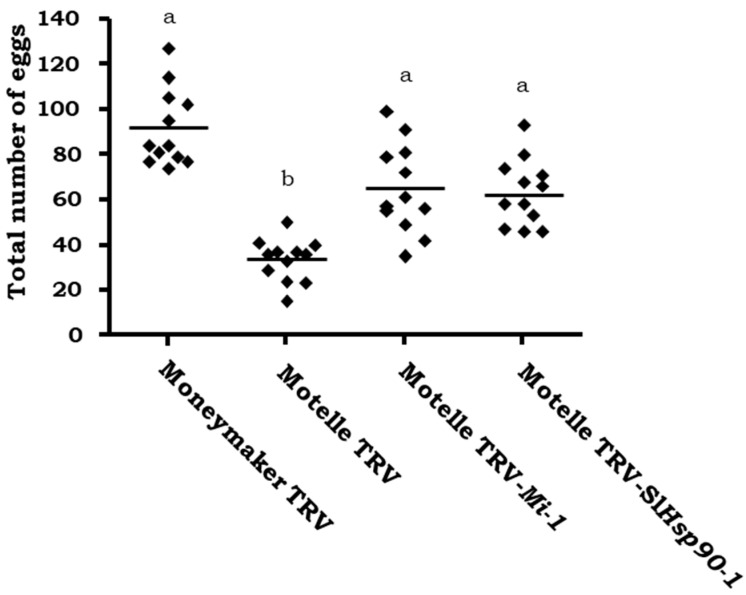
Number of eggs per leaflet on plants of the cultivars Moneymaker *(mi-1/mi-1)* and Motelle *(Mi-1/Mi-1),* agroinfiltrated with the empty pTRV1:pTRV2 vector (TRV), Motelle plants agroinfiltrated with pTRV1:pTRV2-*Mi* (Motelle TRV-*Mi-1*), and Motelle plants agroinfiltrated with the pTRV1:pTRV2-*Hsp90-1* (Motelle TRV-*SlHsp90-1*) and infested with *B. tabaci* females. In each of the three independent assays, four plants were used for each tomato genotype and treatment. Each dot represents the mean number of eggs counted on three leaflets from a single plant. The lines indicate the median values of 12 independent plants. Different letters indicate significant differences (*p* < 0.05) by the non-parametric Kruskal–Wallis test and Dunn’s post-test.

Tomato cultivar Motelle plants were agroinfiltrated with TRV-*SlHsp90-1* and infested with *B. tabaci* in a non-choice assay and whitefly fecundity was evaluated. Plants agroinfiltrated with TRV-*Mi* or empty vector TRV were used as controls. The number of eggs counted on plants agroinfiltrated with TRV-*SlHsp90-1* was significantly higher (*p* < 0.05) than the number on Motelle TRV control plants, and similar to that obtained in plants in which the *Mi-1* gene was silenced (*p* > 0.05) (Figure 7). The Motelle TRV-*Mi* and Motelle TRV-*SlHsp90-1* plants did not show significant differences (*p* > 0.05) when compared with the Moneymaker TRV plants. However, the number of eggs was slightly higher in susceptible plants (Moneymaker TRV) than in Motelle plants agroinfiltrated with TRV-*SlHsp90-1* or TRV-*Mi*.

**Figure 8:**
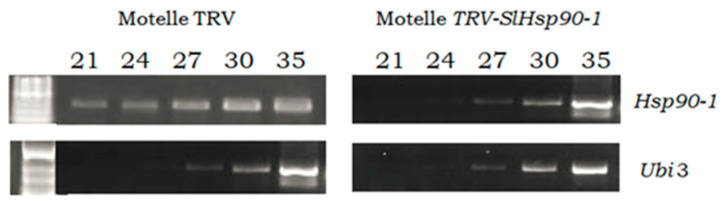
Expression levels of the *Hsp90-1* gene obtained by RT semi-quantitative PCR in leaflets of Motelle plants infiltrated with the empty vector (Motelle TRV) and leaflets of plants agroinfiltrated with agroinfiltrated with the vector pTRV1:pTRV2-*SlHsp90-1* (Motelle TRV-*SlHsp90-1*), both infested with *B. tabaci* females. The ubiquitin gene (*Ubi3*) was used as an endogenous control. The PCR cycles (21, 24, 27, 30, 35) used are indicated.

To confirm the silencing of the *Hsp90-1* gene in TRV-*SlHsp90-1* agroinfiltrated plants, leaflets with a high number of eggs were used in RT semi-quantitative and RT qPCR to evaluate *Hsp90-1* expression. Figure 8 shows the results of the semi-quantitative PCR.

In the control plants (Motelle TRV), the expression of *Hsp90-1* is observed from cycle 21 of the PCR, while in the plants agroinfiltrated with TRV-*SlHsp90-1*, 27 cycles of PCR were needed to obtain a detectable band corresponding to the expression of *Hsp90-1*.

**Figure 9:**
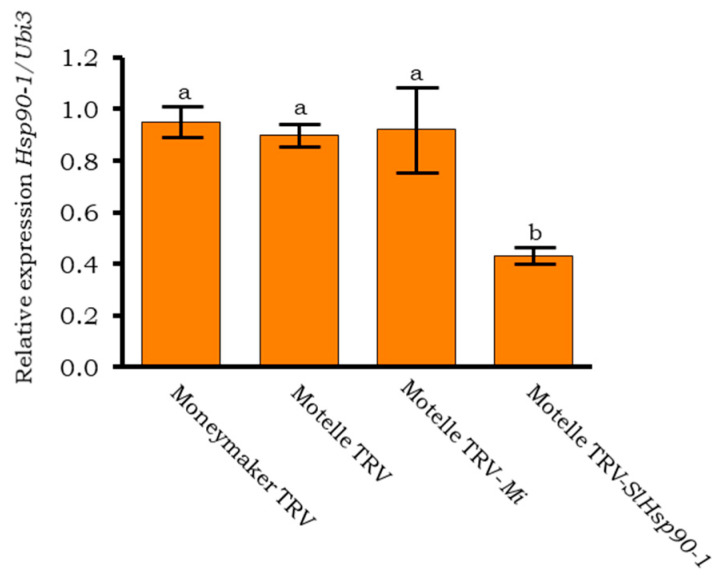
Expression of *HSp-90-1* in agroinfiltrated tomato plants using RT-qPCR analysis. The expression was analysed in leaflets of Motelle and Moneymaker plants agroinfiltrated with pTRV1: pTRV2 empty vector (TRV) and in leaflets of Motelle plants agroinfiltrated with pTRV1:pTRV2-*Mi* (Motelle TRV-*Mi*) or pTRV1:pTRV2-*SlHsp90-1* (Motelle TRV-*SlHsp90-1*), 2 days after infestation with *B. tabaci* females. Each bar is the mean ± standard error of the expression of *Hsp90-1* in two biological samples and three technical replicates, normalized with the expression of the endogenous ubiquitin gene (*Ubi3*). Different letters indicate significant differences (*p* < 0.05) by Tukey’s test.

The quantitative analysis of *Hsp90-1* expression was performed using RT qPCR in the same samples, as well as in Moneymaker TRV plants and Motelle plants agroinfiltrated with TRV-*Mi* (Figure 9).

In the leaflets of the Motelle TRV-*SlHsp90-1* plants, levels of *Hsp90-1* expression were significantly lower (*p* < 0.01) than those observed in the other plants used in this experiment. The expression levels of *Hsp90-1* were similar in Moneymaker and Motelle plants infiltrated with the empty vector TRV, as well as in Motelle plants agroinfiltrated with TRV-*Mi-1*. TRV-*SlHsp90-1* plants showed reduced but not complete suppression of *Hsp90-1* expression. Thus, as for the *Mi-1* gene, *Hsp90-1* silencing occurs partially.

## 3. Discussion

### 3.1. Effects of Agroinfiltration with Empty TRV Vector and TRV-Mi

Our results show that agroinfiltration with the empty TRV vector did not alter basal defence nor *Mi-1*-mediated resistance to *B. tabaci*, as oviposition values were not changed in Moneymaker (*mi-1/-mi-1*) or Motelle (*Mi-1/Mi-1*) plants treated with this vector. Furthermore, the expected differences between the susceptible Moneymaker and resistant Motelle plants were maintained. This supports the suitability of the TRV vector for VIGS studies with whiteflies. These results agree with those obtained with aphids [26] and nematodes [28], confirming that the infiltration of empty TRV does not alter the resistance mediated by the *Mi-1* gene to insects and nematodes. Similarly, TRV infiltration of other plant species did not affect resistance to pathogens mediated by other *R* genes, such as the *N* gene, conferring resistance to tobacco mosaic virus (TMV) [58], or the *SacMi* gene, involved in resistance against *M. incognita* in *Solanum aculeatissimum* [59].

*Mi-1* expression analysis by semi-quantitative PCR of plants agroinfiltrated with the empty TRV vector showed the same pattern previously observed by Li et al. [26] in younger plants of the same cultivars: no *Mi-1* expression was obtained in Moneymaker, while expression in Motelle was observed from cycle 35 of the PCR.

Silencing of the *Mi-1* gene in Motelle plants caused a partial loss of resistance to *B. tabaci*, as has been previously observed for aphids and nematodes using the same TRV vector [26,28]. In Motelle with the *Mi-1* gene silenced, the oviposition of *B. tabaci* females increased compared with non-silenced Motelle and was similar to that of susceptible Moneymaker. This loss of resistance is partial because the silencing by TRV in tomato is not uniform and is patchy throughout the infiltrated leaf, in contrast to the efficiency of VIGS in *Nicotiana benthamiana*, where the silencing is more uniform [41,58,60]. The silencing of the *PDS* gene that produces a visible photobleaching phenotype in the leaves [58] allowed to verify this patchy silencing in the foliar tissues. This patchiness necessitates a larger number of tomato plant replicates for the analysis of any gene whose silencing does not produce a visible phenotype in the plant.

Associated with the partial loss of resistance to *B. tabaci*, semi-quantitative PCR and qPCR analyses confirmed the partial silencing of *Mi-1*, manifested as a significant reduction in the gene expression levels in plants agroinfiltrated with the TRV-*Mi-1* construct.

### 3.2. The Gene Hsp90-1 in the Mi-1-Mediated Resistance to B. tabaci

It was previously known that *Mi-1*-mediated resistance to aphids and nematodes in tomato requires the participation of *Hsp90-1* [28]. Here, our results demonstrate for the first time that the chaperone *Hsp90-1* is also involved in *Mi-1*-mediated resistance to whiteflies. By silencing the *Hsp90-1* gene in the resistant tomato Motelle, a partial loss of resistance to *B. tabaci* was detected, as occurred with the silencing of the *Mi-1* gene in the same tomato cultivar, with very similar oviposition values, significantly higher than those on control Motelle plants. Similarly, *Hsp90* is also involved in the tomato resistance mediated by the *I-2* gene to *Fusarium oxysporum* [46] and in other pathosystems, such as the resistance mediated by the *Pto*, *Rx*, *N*, and *Tm-2^2^* genes to *Pseudomonas syringae* pv. *tomato (Pst)*, potato virus X (PVX), and TMV in *Nicotiana benthamiana* [41,61]. Other examples are the resistance mediated by *RPS2* against *Pst* in *Arabidopsis* [42] or the *PsoRPM2*-mediated resistance to *Meloidogyne incognita* in tobacco [62]. Thus, the requirement of HSP90 seems common in many cases of resistances mediated by plant *R* genes. As HSP90 is a highly conserved protein in most living organisms, involved in many biological processes [31,32,33,34], it could also be relevant for the basal response of plants to damaging organisms. However, the study of the possible involvement of the *Hsp90* gene in the basal response of susceptible tomatoes to *B. tabaci* is outside the scope of the present work.

As noted above for the plants with a silenced *Mi-1* gene, the partial loss of resistance to *B. tabaci* observed in the Motelle plants with silenced *Hsp90-1* was correlated with a significant decrease in the expression levels of this gene, verified by means of semi-quantitative PCR and qPCR. This reduction in *Hsp90-1* expression confirmed the results from a previous work in which the same gene was silenced [28]. Quantitative analysis was also used after VIGS to confirm *Hsp90* silencing in wheat [52], to evaluate the function of SlSERK3A and SlSERK3B in bacterial and nematode innate immunity [63], or to study the roles of the *HsfAs* in *Mi-1.2*-mediated resistance [30].

Previous studies have determined that some chaperones are constitutively expressed, while others are induced in response to stress. *AtHSP90.1* is induced in Arabidopsis by *p. syringae* (*Pst* DC3000) infection, while three other *AtHSP90* isoforms are not [42]. However, during the interaction of tomato/*B. tabaci*, the expression of *Hsp90-1* did not vary in Motelle or Moneymaker plants agroinfiltrated with the empty vector TRV or in plants silenced for *Mi-1*, suggesting constitutive levels for *Hsp90-1* and a regulation independent of *Mi-1*. This invariance in the expression levels of *Hsp90-1* had been observed previously in untreated Motelle plants when comparing non-infested and aphid-infested plants, suggesting that infestation also does not produce variation in the expression levels of *Hsp90-1* [28]. Similarly, previous studies have shown that *Mi-1* expression is also constitutive, with the same level of expression in different organs of the plant, in different developmental stages, and after the attack of nematodes and aphids [64,65]. Although *Hsp90-1* expression is constant, it is known that HSP90 protein levels in tomato are reduced after whitefly infestation [66]. Using polyclonal antibodies, a decrease in HSP90 protein levels has been shown after infestation with both viruliferous (TYLCV-carrying) and non-viruliferous whiteflies, indicating that the stress suffered by the plant translates into a greater consumption of the HSP90 protein, with this reduction in protein levels being more pronounced in plants resistant to TYLCV [66]. Taken together, it could be suggested that the biotic stress caused by infestation with sucking insects in tomato plants does not alter the expression of the *Hsp90* gene, but it does cause a response that reduces the accumulated levels of this protein.

We have observed in this work that *Hsp90-1* silenced plants had a slightly smaller size than the rest of the agroinfiltrated plants. A similar phenomenon has been observed in the silencing of *Hsp90* in *N. benthamiana* [41], suggesting that HSP90 is involved in signalling pathways that affect both plant growth and immunity processes. In wheat too, silencing of certain *Hsp90* genes caused a more pronounced inhibition of wheat seedling growth and even plant death [52].

Several activation models have been described regarding the role of *Hsp90* in *R* gene-mediated resistance [28,47,67]. In *Mi-1*-mediated resistance, the model based on the “gatekeeper hypothesis” [68] proposes an interaction between Mi-1, HSP90, and SGT1 proteins that “guard” the RME1 protein [28]. Previous works had already shown that *Rme1* is required for *Mi-1*-mediated resistance to nematodes, aphids, and whiteflies [25,29], but not for *I-2* gene-mediated resistance to *F. oxysporum* f.sp. *lycopersici* [29] or for *Pto*-mediated resistance to *Pst* [25]. In the model proposed by Bhattarai et al. (2007) [28] for *Mi-1* gene-mediated resistance to aphids and nematodes, HSP90 interacts with SGT1 but not RAR1, and RME1 is the target protein for nematode and insect avirulence proteins, which would modify RME1. This modification would cause a conformational change in the Mi-1 protein that would allow the union of HSP90 and SGT1, to form a signalosome that would activate the signalling pathway and defence in the plant. Our previous and current results with *B. tabaci* reinforce the hypothesis of a common model for nematodes and insects in the interaction of Mi-1, RME1, and HSP90 proteins. From this point on, further evaluation of the expression of other marker genes downstream to *Mi-1* will allow to deepen the knowledge on the entire pathway in the complex process of the *Mi-1*-mediated resistance of tomato against nematodes, aphids, and whiteflies.

## 4. Materials and Methods

### 4.1. Plant Material and Growth Conditions

**Figure 10:**
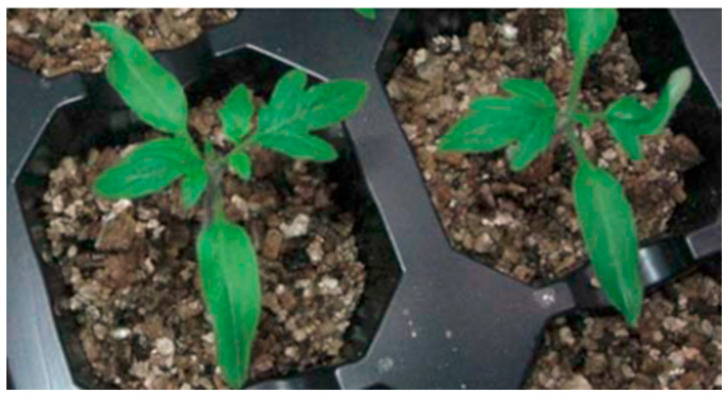
Tomato seedlings grown at 24 °C/20 °C (L/D), with two true leaves, ready for infiltration.

Tomato (*Solanum lycopersicum* L.) near isogenic lines Motelle and Moneymaker were used in this study. Motelle is homozygous dominant for the *Mi-1 R* gene (*Mi-1*/*Mi-1*) and Moneymaker is homozygous recessive (*mi-1*/*mi-1*). These cultivars differ only in the presence of a 650 kb introgressed region from *Solanum peruvianum* containing the *Mi-1* gene, in chromosome 6 of Motelle [69,70]. Seeds were germinated in sterile vermiculite (number 3, Projar, Spain) inside a growth chamber under controlled conditions: 24 °C and 20 °C (16 h day/8 h night) at 70% relative humidity. Plants were watered when needed and supplied every 15 days with a 20/20/20 nutritive complex (Nutrichem 60; Miller Chemical, Hanover, PA, USA) at 3 g·L^−1^. Approximately 10 days after germination, when the first true leaves began to emerge, cultivation of the agro clones was started, as described below (see Section 4.3). Around 15 days after germination, the first two true leaves had generally already developed (Figure 10) and agroinfiltration of these leaves and cotyledons was carried out. During and after infiltration, care was always taken to keep the plants in separate trays for each genotype and treatment, in order to avoid possible accidental transmission of the virus by contact or during irrigation. About 8 weeks after infiltration, bioassays were carried out by infesting the plants with whiteflies.

### 4.2. VIGS Constructs

The Tobacco Rattle Virus (TRV) vector was used for gene silencing. It is made up of pTRV1 (TRV1), which contains the replicative part of the virus, and pTRV2 (TRV2), in which the gene to be silenced is inserted. This vector has been extensively described [58].

The pTRV2-*SlHsp90-1*, pTRV2-*Mi-1*, and pTRV2-*PDS* constructs were used to silence the *Hsp90-1*, *Mi-1,* and *PDS* genes, respectively. Moreover, an empty pTRV2, without introduced genetic material, was also used to check if the infiltration itself produces any alteration in the behaviour of *B. tabaci*. The silencing of *Mi-1* has been carried out successfully before [26], but its effect on resistance to whiteflies has not been studied. The silencing of the *PDS* gene aimed to evaluate the efficacy of the silencing, as it produces a visible phenotype of photobleaching due to the suppression of the activity of phytoene desaturase, which participates in the biosynthesis of carotenoids [26,58].

The pTRV1:pTRV2-*PDS* (TRV-*PDS*) and pTRV1:pTRV2-*SlHsp90-1* (TRV-*SlHsp90-1*) constructs were obtained from Dr. S. Dinesh Kumar [35,58]. The pTRV1:pTRV2-*Mi-1* (TRV-*Mi-1*) construction was carried out by cloning a 300 bp fragment of the *Mi-1* cDNA and using the primers C1/2Do and C2S4 for their amplification [66]. The amplified fragment was cloned into pGEM T-Easy vector and restricted using SphI and then treated with T4 DNA polymerase. The resulting fragment was digested with SacI and introduced into pTRV2. The plasmid formed was transformed into a culture of *Agrobacterium tumefaciens* GV3101. Each of the agroclones (TRV1 and TRV2) obtained came from an isolated colony and were preserved in glycerol at −80 °C.

### 4.3. Growth of Agroclones and Agroinfiltration

As mentioned above, the growth of agroclones started approximately 10 days after germination of tomato seeds. Firstly, agroclones were grown on Petri dishes with 1.5% agar in LB medium supplemented with 50 mg·L^−1^ Kanamycin and 25 mg·L^−1^ Rifampicin at 28 °C for 24–48 h. Two days before the agroinfiltration, an isolated colony of each agroclone was introduced into a test tube containing 2 mL of LB medium supplemented with 50 mg·L^−1^ Kanamycin and 25 mg·L^−1^ and incubated overnight at 28 °C with horizontal agitation (200–250 rpm). Then, 2 ml of each culture was added to a 125 mL Erlenmeyer flask containing 25 mL of LB medium supplemented with Kanamycin (50 mg·L^−1^), Rifampicin (25 mg·L^−1^), Acetosyringone (20 µM), and MES (10 mM). Erlenmeyer flasks were incubated overnight at 28 °C with horizontal agitation (200–250 rpm).

The day of agroinfiltration (approximately 15 days after germination, as mentioned at the beginning) depended on whether the plants had already developed their first two true leaves. This day, the cultures were centrifuged at 2800 rpm at room temperature for 10 min. The pellet was resuspended in a freshly prepared infiltration buffer (10 mM MgCl_2_, 10 mM MES, and 200 µM Acetosyringone). The concentration was measured in a spectrophotometer at OD_600nm_ and adjusted to an absorbance value of 2. Then, the agroclones were gently stirred for 3–5 h in the dark. After this incubation period, the leaves were infiltrated as follows.

**Figure 11:**
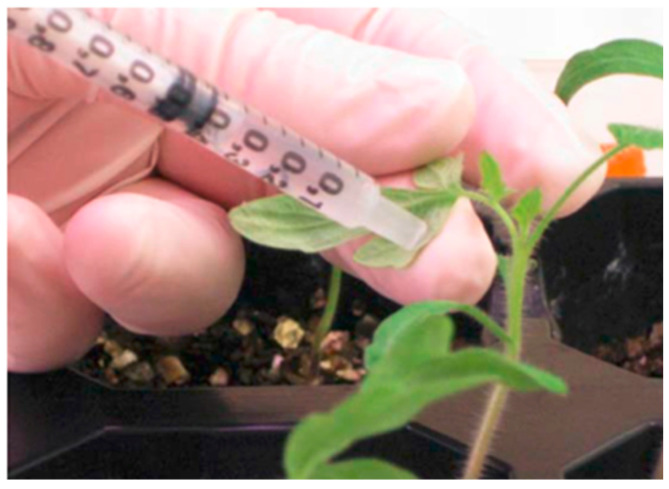
Agroinfiltration of tomato seedlings in the underside of leaves, using a needleless syringe for virus-induced gene silencing.

Firstly, TRV1 was mixed with each TRV2 (‘empty’ or with the gene of interest to be silenced) in a 1:1 ratio to obtain a total volume of 8 mL, usually enough to infiltrate 10 plants. The mixture was aspirated with a needleless syringe and the infiltration was carried out by gently pressing with the syringe on the underside of the leaves and cotyledons so as not to cause any mechanical damage to the plants (Figure 11).

Ten days after infiltration, plants were ready for transplanting into pots. The temperature at infiltration was reduced to 19 °C and maintained during the next 8 weeks to delay plant development, thus favouring the virus distribution. So, these 10-week-old plants reached a degree of development similar to that of 8-week-old plants that had grown at 24 °C and 20 °C (day/night), which is important as tomato resistance mediated by *Mi-1* to *B. tabaci* is dependent on plant development [71].

### 4.4. Bioassays with Plants Infested with B. tabaci

Moneymaker and Motelle plants were used, both uninfiltrated and infiltrated with pTRV1:pTRV2-’empty’ (TRV). In addition, Motelle plants infiltrated with pTRV1:pTRV2-*Mi-1* (TRV-*Mi-1*) and with pTRV1:pTRV2-*SlHsp90-1* (TRV-*SlHsp90-1*) were also used. These six types of plants were infested in non-choice bioassays with adult females of the MED (Mediterranean species) *B. tabaci*. A population of these whiteflies was reared for several generations in our laboratory, free from any plant pathogen, on the susceptible tomato cultivar Marmande. Infestation was performed inside a growth chamber at 24 °C and 20 °C (16 h day/8 h night) at 70% relative humidity. Briefly, each of three well-developed leaflets from the upper part of the tomato plant was placed into a cage made up from a 50 mL Falcon tube following the methodology described in Rodriguez et al. [23]. Twenty female whiteflies were taken from the rearing colony by vacuum aspiration and deposited into each tube containing a leaflet. Four replicated plants were used for each plant genotype and treatment and three independent assays were carried out. After two days, the tubes and the whiteflies were removed and the number of eggs on each leaflet was counted. The statistical analysis of the data was performed by the non-parametric Kruskal–Wallis test and Dunn’s post-test, as more than two groups were compared.

Immediately after egg counting, infested leaflets were cut with a scalpel and placed individually in Eppendorf tubes containing glass beads, frozen in liquid nitrogen, and stored at −80 °C until RNA extraction for gene expression analyses. In addition, other non-infested leaflets were collected from the same plants, individually placed in hermetically sealed plastic bags, and kept on ice until they were used to extract genomic DNA in order to confirm the plant genotype in terms of the presence/absence of the *Mi-1* gene.

### 4.5. Genomic DNA (gDNA) Extraction and PCR Amplification

Genomic DNA was extracted from three young leaflets from each plant, following the protocol described by Peterson et al. [72] with slight modifications. The extracted genomic DNA was quantified prior to amplification. For PCR, 50–100 ng of DNA was used and the amplification was carried out in a programmable thermal cycler Thermal Blok II^®^ (Lab-Line Instruments^®^, Dubuque, IA, USA). The reaction was as follows: 3 min at 94 °C, followed by 30 cycles of three steps each: 30 s at 94 °C, 30 s at 64 °C, and 30 s at 72 °C. Finally, a cycle of 5 min at 72 °C. The amplification of *Mi-1* from gDNA was performed using the primers PMiF3 (5′-GGTATGAGCATGCTTAATCAGAGCTCTC-3′) and PMiR3 (5′-CCTACAAGAAATTATTGTGCGTGTGAATG-3′), designed by El Mehrach et al. [73].

### 4.6. RNA Extraction and cDNA Synthesis and Amplification

Total RNA was extracted from the leaflets collected in the *B. tabaci* infestation bioassays. Total RNA extraction was performed using Trizol (Invitrogen, Waltham, MA, USA), in a three-step process, including (1) homogenization, separation, precipitation, washing, and dissolution; (2) treatment with DNase RQI (Promega); and (3) re-extraction of the RNA using phenol/chloroform.

cDNA synthesis was performed using 5 µg of the total RNA extracted, which was reverse-transcribed using Superscript II reverse transcriptase (Invitrogen). The synthesized cDNA was amplified by PCR for semi-quantitative analysis and those samples with greater interest were subsequently amplified by qRT-PCR (qPCR). The semi-quantitative PCR amplification consisted of 5 min at 95 °C for a variable number of three-step cycles: 45 s at 94 °C, 45 s at 60 °C, and 1 min at 72 °C. For the amplification of *Mi-1*, 35, 40, 45, and 50 cycles were performed [26], while for the amplification of *Hsp90-1*, 21, 24, 27, 30, and 35 cycles were used [28]. The expression of the endogenous *Ubi3* gene, which has been used in previous VIGS works [26,28], was used as a control. The amplified products were analysed by electrophoresis on Ethidium bromide stained with 1.5% (*w*/*v*) agarose gels.

By this semi-quantitative PCR, the cDNA extracted from leaflets of Moneymaker and Motelle plants infiltrated with the empty vector (pTRV2-’empty’) was amplified. cDNA was also amplified from Motelle plants silenced for the *Mi-1* gene or for the *Hsp90* gene, selecting those leaflets in which the number of *B. tabaci* eggs was higher than that observed in Motelle plants not silenced but infiltrated with the empty vector. The sequences of primers used are detailed in previous works [26,28].

The most interesting samples amplified by semi-quantitative PCR, which confirmed the silencing of the genes, were quantified using real-time quantitative PCR (qRT-PCR or qPCR). This procedure measures the concentration of cDNA in a sample, using a probe that emits fluorescence (SYBR Green^®^, Alameda, CA, USA) when bound to nucleic acid. The quantification of the samples was carried out in the 7900HT Fast Real Time PCR system (Applied Biosystems, Waltham, MA, USA) of the Genomics Service of the “Albert Sols” Institute (http://www.iib.uam.es/portal/web/genomica), and *Ubiquitin* was used as an endogenous control gene for quantification. Two biological samples (leaflets) per plant type were used and each of the qPCR reactions was performed in triplicate for each biological sample. Leaflets of *Mi-1* or *Hsp90* silenced Motelle plants, in which the number of *B. tabaci* eggs was higher than that in Motelle leaflets of not silenced plants but infiltrated with the empty vector (pTRV2-‘empty’), were analysed. Leaflets from Moneymaker plants were also tested as controls. The qPCR conditions were 10 min at 95 °C, followed by 40 cycles of 15 s at 95 °C and 1 min at 60 °C. Finally, the dissociation curve was carried out, for 15 s at 95 °C, followed by 15 s at 60 °C and finally 15 s at 95 °C. The primers used in this study were specifically designed using the Primer Express^®^ program (Applied Biosystems) and their specificity was checked against the SGN and NCBI databases. The primers are detailed in Table 1. The relative expression of each gene was calculated using the ∆∆CT method, comparing the data with the reference gene (*Ubi3*). Finally, the mean of the triplicates and the associated error were calculated. The data obtained were analyzed using the Tukey test to compare all of the samples with each other.

## Figures and Tables

**Table 1:** Primers designed for qRT-PCR of *Mi-1*, *Hsp90-1*, and *Ubi3* genes.

Gene	Primer name	Sequence
*Mi-1*	Mi-RT-F	5′-AGAGGAGGGAACGATCTTCAGA-3′
	Mi-RT-R	5′-AAGCAAAGTTCAACCAAAATGCT-3′
*Hsp90-1*	HSP90-RT-F	5′-TAGCCTTGATGAGCCAAACACA-3′
	HSP90-RT-R	5′-CGATACTCAGACCAAGCTTCAGC-3′
*Ubi3*	Ubi3-RT-F	5′-TGTGGGCTCACCTACGTTTACA-3′
	Ubi3-RT-R	5′-CTGATAGAGCATTGCTAAACATTAAAATC-3′

## Data Availability

The data presented in this study are available on request from the corresponding author.
